# Feto-maternal biology and ethics of human society

**DOI:** 10.1186/1477-7827-3-55

**Published:** 2005-10-18

**Authors:** Luana Paulesu, Francesca Ietta, Felice Petraglia

**Affiliations:** 1 Department of Physiology, Division of Immunoendocrinology and Reproductive Physiology, University of Siena, 53100 Siena, Italy; 2 Department of Pediatrics, Obstetrics and Reproductive Medicine, University of Siena, 53100 Siena, Italy

## Abstract

The growing interest in human reproduction and the identity of the embryo have prompted us to bring some considerations to the attention of scientists. In particular, we focus on the interactive relationship between the embryo and the mother starting from the earliest stages of development. Principles governing the acceptance and growth of the embryo in the uterus may represent a model for mutual tolerance and peaceful co-existence in human society.

## The fetus as an allograft

The embryo is a semi-allograft in the maternal organism because half of its genetic material comes from the father. However, instead of being rejected by the maternal immune system, it is tolerated and develops in the uterus [1].

During early embryogenesis, the trophoblast of the external layer of the morula-blastocyst makes direct contact with the uterine wall (blastocyst implantation). In human pregnancy, the trophoblast invades the uterine mucosa and vessels, establishing very intimate contact with the mother (hemochorial placenta). Therefore, the trophoblast and maternal uterus, including immune and non-immune cells of the mucosa and vessels, form the feto-maternal interface, in which tolerance mechanisms are active [2].

## Feto-maternal dialogue

Ever since Sir Peter Medawar brought the topic of survival of the semi-allogenic fetus in the maternal uterus to the attention of scientists, various studies have sought to throw light on the biochemical and molecular mechanisms that permit this apparent immunological paradox [3,4]. A key role has been attributed to the secretion of a broad array of soluble molecules with autocrine/paracrine action, including growth factors, cytokines and hormones [5]. These substances are produced at the feto-maternal interface by both embryonic and maternal tissues, and they act on specific membrane receptors expressed by complementary tissues [6]. Thus, mother and embryo interact via specific tissues (trophoblast and uterus) in a reciprocal exchange of molecules that act as communication signals. This interactive relationship between the embryo and the mother has the characteristics of a true dialogue: a dialogue that uses molecules instead of words and that takes place in a common language, comprehensible to both mother and embryo.

The feto-maternal dialogue begins very early in embryonic development, initiated by the trophoblast via secretion of molecules such as hCG which act on the mother to create a uterine environment favorable to implantation [7]. The uterus responds to these embryonic signals with molecules that favor the survival and growth of the trophoblast instead of rejecting it [8]. It is noteworthy that, during the normal menstrual cycle, the uterine mucosa undergoes changes that make it receptive to implantation [9]. These changes reach their maximal expression during the luteal phase "window of implantation" when, embryonic signals, if present, become effective. Thus, the establishment of the feto-maternal dialogue requires the synchrony and active contribution of both partners, otherwise the signals sent by the embryo would be left unheard.

The feto-maternal dialogue continues throughout pregnancy, developing different languages (complexes of molecules) and tones (concentrations of molecules) according to the phases of gestation. Thus, while pro-inflammatory cytokines 1 and Th1 cytokines are prevalent during implantation and labor, anti-inflammatory and Th2 cytokines are predominant during the maintenance of pregnancy [8,10].

Although the exact languages used in the feto-maternal dialogue have yet to be completely deciphered, it is known that a correct molecular dialogue between mother and fetus is fundamental for the health of both: a lack of (or alterations to) this dialogue may result in early abortion or poor placentation leading to retarded growth or death for the fetus and to hypertensive disturbances or other gestational diseases for the mother [11].

## Ethical implications for human society

Biological principles may represent a model for human society, which has always faced the impact of "diversity": indeed, history is full of interactions between different populations, cultures and religions.

Embryonic development and adaptation require a correct dialogue with the recipient, so that the diversity of the "other" is not rejected but, instead, reciprocally welcomed. This dialogue is based on recognition, not suppression, of the identity of the other, reciprocity and receptivity. It consists of molecules (words) that, instead of offending, prepare the other for a response that benefits mutual well-being.

Therefore, the biological mechanisms underlying feto-maternal relationships teach us that diversities should not be opposed; their co-existence is possible, indeed fundamental for life. These mechanisms also suggest that a correct dialogue with someone "different" leads to tolerance as well as growth and development. These biological principles, embedded in the reality of life, can help us to seek an equilibrium of social relationships. Otherwise, diversity causes only discord, death and destruction.
